# Inflation, Economic Policy Changes, and Access to Essential Drugs by Retirees in Argentina

**DOI:** 10.1001/jamanetworkopen.2024.15929

**Published:** 2024-06-10

**Authors:** Alejandro Macchia, Javier A. Mariani, Gianni Tognoni

**Affiliations:** 1 Fundación GESICA (Grupo de Estudio Sobre Investigación Clínica en Argentina), Buenos Aires, Argentina; 2 The Permanent Peoples’ Tribunal, Rome, Italy

## Abstract

This cross-sectional study investigates whether there are associations between inflation, economic policy changes, and retirees’ access to medicines in Argentina.

## Introduction

Access to medicines, integral to health rights, faces a global, chronic, and multicausal gap between declared rights and actual availability, particularly affecting at-risk groups.^1,2^ In Argentina, the disparity is pronounced with two-thirds of retirees living on minimal pensions. Recent economic policies have exacerbated challenges by deregulating drug prices, following a 100% devaluation of the peso against the US dollar. Previously, drug price increases required government approval, but recent shifts have led to a deregulated pharmaceutical market, allowing companies to set prices freely. This study explores the associations between inflation, economic policy changes, and retirees’ access to medicines.

## Methods

In this cross-sectional study, we tracked prices and affordability of 360 essential drugs from December 2020 to January 2024. These essential drugs are mainly synthetic, nonbiological, and often not patent-protected, deemed essential by the Buenos Aires Government for its health care facilities. Only drugs consistently available throughout the 38-month period were included, with cumulative price increases calculated against December 2020 prices, expressed as a percentage. Drug prices are periodically published on the Alfabeta and Kairosweb portals and are publicly accessible.

Inflation rates from the National Institute of Statistics and Censuses and pension data from the National Administration of Social Security provided contextual economic and social security insights. Argentina’s pension scheme requires a minimum of 30 years of contributions for eligibility at 60 years of age for women and 65 years of age for men. Monthly pensions, often not aligned with inflation, are commonly supplemented with a bonus payment. As of January 2024, more than two-thirds of Argentine retirees, with a mean age of 71.7 years, received the minimum pension, totaling $93 USD or $142 USD with the bonus. In addition, the study assessed the financial burden of cardiovascular medication on minimum pension incomes; a standard treatment regimen consisting of a β-blocker, a low-dose aspirin, and a statin was selected for analysis.

This study did not require ethical review nor informed consent per national guidelines in Argentina (resolution No. 1480-11). We followed the STROBE reporting guideline. Statistical analyses were performed using RStudio version 2023.12.1 (R Project for Statistical Computing) from January to February 2024.

## Results

**Figure 1.**
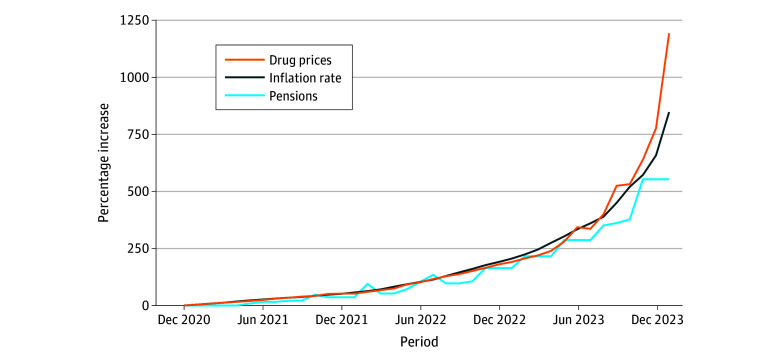
Rising Cost of Medicines, Pensions, and Inflation in Argentina, 2020-2024

**Figure 2.**
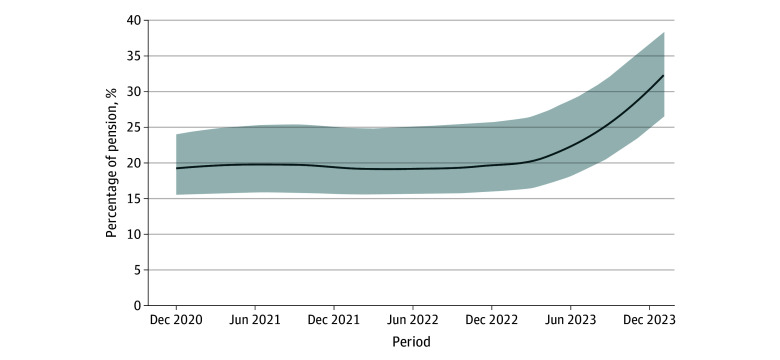
Percentage of Pension Benefits Required to Obtain a Month’s Treatment With 3 Essential Drugs^a^ ^a ^The 3 essential drugs were for a basic cardiovascular treatment regimen and consisted of a β-blocker, a low-dose aspirin, and a statin.

Among 360 essential drugs tracked between December 2020 and January 2024, essential drug prices in Argentina increased a median (IQR) of 1051% (923%-1174%), surpassing the cumulative inflation of 849%, with a notable acceleration in price increases from October 2023 after the national election. Pension benefits had increased by 455% based on the standard adjustment formula and by 744% when including exceptional bonuses (Figure 1). The cost burden of cardiovascular medications on minimum pensions also increased sharply; by January 2024, retirees needed to allocate 34.9% of their pension for a basic treatment regimen of 3 drugs, up from 17.4% in December 2020 (Figure 2). Uninsured patients faced the full retail price, while private insurance typically covered approximately 40% of these costs, leaving patients to pay the remaining 60%. For those with social insurance, including the public health care program for retirees (PAMI), out-of-pocket costs fluctuated, with subsidies covering between 30% to 70% of medication expenses. Additionally, PAMI offers 167 essential medications free of charge to its members.

## Discussion

In this study, the liberalization of drug prices was associated with increased financial pressure on retirees, meaning they would have to spend more than a third of their pensions on a basic treatment regimen and approaching the widely accepted benchmark of substantial health expenditure.^3^ Currency devaluation and deregulation of drug prices were associated with a substantial increase in drug costs, exceeding the rate of inflation and pension increases, potentially affecting individuals who are financially at risk.^4,5^ Limitations of the study include the restricted list of medicines analyzed and the variability of the effect of health coverage, which highlight broader health equity issues.

## Notes

Supplement.
Data Sharing Statement
