# Supplementary material for: Inflation, Economic Policy Changes, and Access to Essential Drugs by Retirees in Argentina
Source: JAMA Netw Open. 2024 Jun 10;7(6):e2415929. doi: 10.1001/jamanetworkopen.2024.15929 (PMC11165379; doi:10.1001/jamanetworkopen.2024.15929)
Supplement: Supplement. — Data Sharing Statement [file jamanetwopen-e2415929-s001.pdf]

Data Sharing Statement

Macchia. Inflation, Economic Policy Changes, and Access to Essential Drugs by Retirees in Argentina. *JAMA Netw Open*. Published June 10, 2024.
doi:10.1001/jamanetworkopen.2024.15929

Data
Data available: No
